# Patient-derived glioblastoma cell lines with conserved genome profiles of the original tissue

**DOI:** 10.1038/s41597-023-02365-y

**Published:** 2023-07-12

**Authors:** Soon-Chan Kim, Young-Eun Cho, Young-Kyoung Shin, Hyeon Jong Yu, Tamrin Chowdhury, Sojin Kim, Kyung Sik Yi, Chi-Hoon Choi, Sang-Hoon Cha, Chul-Kee Park, Ja-Lok Ku

**Affiliations:** 1 Korean Cell Line Bank, Laboratory of Cell Biology, Cancer Research Institute, Seoul National University College of Medicine, Seoul, 03080 Republic of Korea; 2 Cancer Research Institute, Seoul National University, Seoul, 03080 Republic of Korea; 3 Ischemic/Hypoxic Disease Institute, Seoul National University College of Medicine, Seoul, 03080 Republic of Korea; 4 Department of Neurosurgery, Seoul National University Hospital and Seoul National University College of Medicine, Seoul, 03080 Republic of Korea; 5 Department of Radiology, Chungbuk National University Hospital and Chungbuk National University College of Medicine, Cheongju, Chung Buk 28644 Republic of Korea; 6 Chungbuk National University College of Medicine, Cheongju, Chung Buk 28644 Republic of Korea; 7 Department of Biomedical Sciences, Seoul National University College of Medicine, Seoul, 03080 Korea

**Keywords:** Genetic databases, CNS cancer

## Abstract

Glioblastoma (GBM) is the most lethal intracranial tumor. Sequencing technologies have supported personalized therapy for precise diagnosis and optimal treatment of GBM by revealing clinically actionable molecular characteristics. Although accumulating sequence data from brain tumors and matched normal tissues have facilitated a comprehensive understanding of genomic features of GBM, these in silico evaluations could gain more biological credibility when they are verified with *in vitro* and *in vivo* models. From this perspective, GBM cell lines with whole exome sequencing (WES) datasets of matched tumor tissues and normal blood are suitable biological platforms to not only investigate molecular markers of GBM but also validate the applicability of druggable targets. Here, we provide a complete WES dataset of 26 GBM patient-derived cell lines along with their matched tumor tissues and blood to demonstrate that cell lines can mostly recapitulate genomic profiles of original tumors such as mutational signatures and copy number alterations.

## Backgrounds & Summary

Glioblastoma (GBM) is one of the most aggressive forms of malignancies. Due to its aggressiveness and molecular complexity, the prognosis of GBM patients has not been improved compared to patients with other types of cancer^1^. With advanced sequencing technologies, massive sequencing of brain tumor tissues has improved our understanding of genomic characteristics of GBM^2,3^. Nevertheless, unmatched sequencing results between tumor tissues and *in vitro* models still kept the in silico molecular comprehensions from pre-clinical applications such as high-throughput screening (HTS)^4^. In an effort to close the gap between in silico analysis and actual biological models, broad institute cancer cell lines encyclopedia (CCLE) project have made significant progress to thoroughly analyze omics data of widely used cancer cell lines^5^. Sixty-six glioma cell lines were massively analyzed in the CCLE project, yet the molecular and physiological resemblances between parental tumor tissues and cell lines remain uncertain. Here, we provide a complete set of whole exome sequencing (WES) from successfully established patient-derived cell lines along with their matched tissues and bloods of 26 different GBM patients. Although sequencing data from either GBM tissues or cell lines has been massively deposited, there are only few databases encompassing both tissues and cell lines with matched normal. Our data indicated that GBM cell lines recapitulated representative pathogenic mutations of the original tumor and the germline mutations were exclusively present in matched blood DNA. All cell lines introduced in this study including its genomic profiles will be deposited to Korean Cell Line Bank (http://cellbank.snu.ac.kr) at initial passages to be distributed to researchers worldwide.

**Fig. 1:**
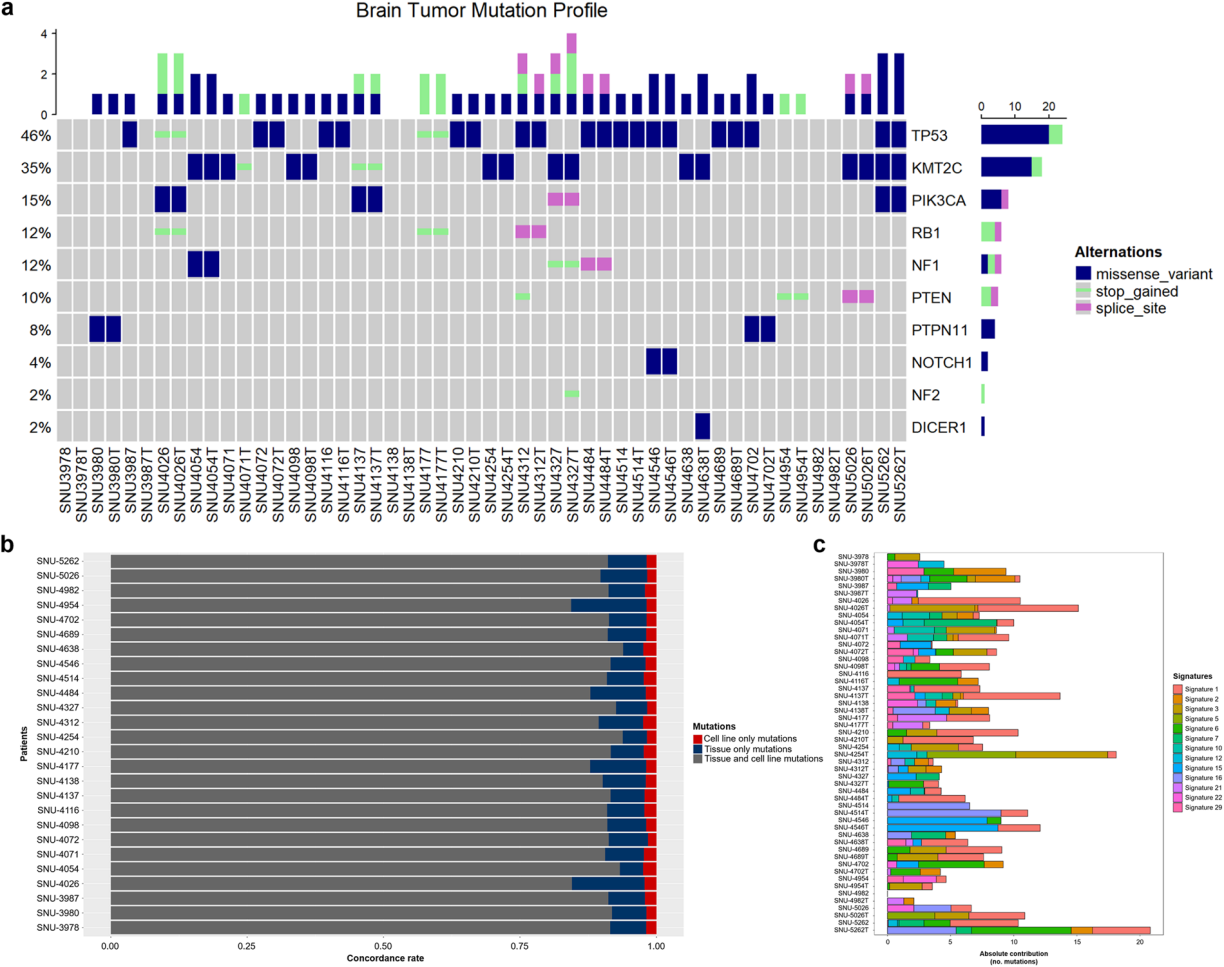
(**a**) Waterfall plot of mutations indicates that majority of potential driver mutations were exclusively present at the tissue and cell lines. Frequently mutated genes were *TP53*, *NF1* and *PTEN*. (**b**) Concordance plot designates that the established cell lines mostly recapitulated the mutational landscapes of the original tumors. (**c**) Mutational signature analysis displayed the characteristic composition of mutational signature in the parental tissues was maintained in the cell lines.

WES identified several mutations in the applied samples, including point mutations in putative oncogenes. We have excluded benign mutations by referring sequencing results from patient blood DNA and Clinvar database^6^. Mutations commonly observed in Glioblastoma^3,7^ were well presented in our cell lines. These include inactivating mutations in tumor suppressors such as *TP53* and *PTEN* as well as activating alterations in the *PIK3CA*. No representative driver mutation was detected in the SNU-3978 cell line (Fig. 1a). Missense mutations at the promotor regions of *TERT* has been associated with increased telomerase activity and eventually malignant tumors including Glioblastoma^8,9^. Since promotor regions were mostly uncovered by WES, we manually verified *TERT* promoter mutations among 26 GBM cancer cell lines using Sanger sequencing (Table 1). SNU-3978 harbored C228T missense mutation at the *TERT*, which might function as a driver mutation. Lollipop plot of *TP53* indicates that the nonsense mutation pointed by blue arrows exclusively presented at the tissue and cell line samples, which implied these are the potential driver mutations. The missense mutation and nonsense mutations highlighted by red arrows were harbored by cell lines only (Supplementary Fig. 1a). For instance, SNU-4098 had a pathogenic mutation at *TP53* (c.273 G > A, Trp91Ter) which was undetected in its parental tissue samples (Fig. 1a). This incongruity between the original tissues and cell lines might be caused by both acquisition of random somatic mutations during passaging or low cellularity of the tissue sample.

**Table 1:** Clinicopathological information of 26 glioblastoma patients.

SNU No	Sex	Age	Location	Histological Diagnosis	WHO grade	IDH1/2 (Sanger sequencing)	ATRX (Immunohistochemistry)	Tissue TERT promoter mutation	Cell Line TERT promotor mutation	MGMT promoter	1p/19q (FISH)	7p12 (FISH)	9p21 (FISH)	10q23 (FISH)
SNU-3978	M	49	right temporal	Glioblastoma	4	WT	positive	C228T	C228T, T349C	unmethylation	Nodel	no amplification	Nodel	Del(Hemi)
SNU-3980	M	49	left hippocampus	Glioblastoma	4	WT	positive	C228T	C228T	methylation	Nodel	no amplification	Del(Hemi)	Nodel
SNU-3987	M	60	right temporal	Glioblastoma	4	WT	positive	WT	C228T, C348T	methylation	Nodel	no amplification	Nodel	Nodel
SNU-4026	M	65	left temporal	Glioblastoma	4	WT	positive	C228T	C228T	unmethylation	Nodel	no amplification	Nodel	Nodel
SNU-4054	M	65	right frontal	Glioblastoma	4	WT	positive	WT	WT	methylation	1pDel	no amplification	Del(Homo)	Nodel
SNU-4071	M	57	left temporal	Glioblastoma	4	WT	positive	C250T	C250T	unmethylation	Nodel	no amplification	Del(Homo-Hemi)	Del(Homo-Hemi)
SNU-4072	M	72	right insulo-temporal	Glioblastoma	4	WT	positive	C228T	C228T	unmethylation	Nodel	amplification	Del(Homo)	Nodel
SNU-4098	F	74	left temporal	Glioblastoma	4	WT	positive	C228T	C228T	unmethylation	Nodel	amplification	Del(Homo-Hemi)	Nodel
SNU-4116	F	57	left parieto-temporal	Glioblastoma	4	WT	positive	C228T	C228T	unmethylation	Nodel	amplification	Del(Homo)	Nodel
SNU-4137	M	80	right frontal	Glioblastoma	4	WT	positive	C228T	C228T	unmethylation	Nodel	amplification	Del(Homo)	Del(Hemi)
SNU-4138	F	75	right temporal	Glioblastoma	4	WT	positive	WT	T349C	methylation	Nodel	no amplification	Del(Homo)	Nodel
SNU-4177	F	68	left temporal	Glioblastoma	4	WT	negative	C228T	C228T	unmethylation	Nodel	no amplification	Nodel	Del(Hemi)
SNU-4210	F	79	left frontal	Glioblastoma	4	WT	positive	C250T	C250T	methylation	Nodel	amplification	Del(Homo)	Nodel
SNU-4254	M	61	left temporal	Glioblastoma	4	WT	positive	WT	WT	unmethylation	19qDel	no amplification	Del(Homo)	Nodel
SNU-4312	F	65	left hemispheric	Glioblastoma	4	WT	positive	C228T	C228T, T348C	unmethylation	Nodel	amplification	Del(Homo-Hemi)	Nodel
SNU-4327	M	69	left temporal	Glioblastoma	4	WT	positive	C228T	C228T, T348C	methylation	Nodel	no amplification	Del(Homo)	Nodel
SNU-4484	M	66	right hemispheric	Glioblastoma	4	WT	positive	C228T	C228T, T349C	methylation	Nodel	no amplification	Del(Hemi)	Del(Homo)
SNU-4514	M	67	right frontal	Glioblastoma	4	WT	positive	C228T	C228T, T349C	unmethylation	Nodel	no amplification	Nodel	Nodel
SNU-4546	F	54	bi-frontal (butterfly)	Glioblastoma	4	WT	positive	C250T	C250T	methylation	Nodel	no amplification	Nodel	Del(Homo-Hemi)
SNU-4638	F	56	left fronto-insulo-temporal	Glioblastoma	4	WT	positive	WT	WT	Unmethylation	Nodel	amplification	Nodel	Nodel
SNU-4689	M	77	right frontal	Glioblastoma	4	WT	positive	C250T	C250T	methylation	Nodel	no amplification	Nodel	Nodel
SNU-4702	M	75	right fronto-insulo-temporal	Glioblastoma	4	WT	positive	C228T	C228T, T349C	methylation	Nodel	no amplification	Nodel	Del(Hemi)
SNU-4954	F	31	right frontal	Glioblastoma	4	WT	positive	WT	T349C	Unmethylation	Nodel	no amplification	Nodel	Nodel
SNU-4982	M	56	left fronto-parietal	Glioblastoma	4	WT	negative	WT	WT	methylation	Nodel	no amplification	Nodel	Nodel
SNU-5026	M	31	left fronto-insulo-temporal	Glioblastoma	4	WT	positive	WT	WT	Unmethylation	Nodel	no amplification	Nodel	Nodel
SNU-5262	F	66	right temporal	Glioblastoma	4	WT	positive	C228T	C228T	methylation	19qDel	no amplification	Nodel	Nodel

Mutational concordances within the coding regions between the original tumor tissues and cell lines indicated that cell lines well recapitulated the mutational trait of the matched tumor specimens (median = 0.91 frequency of concordance ranging 0.84 to 0.94). While the portion of cell line-specific mutations were analogous to each other (median = 0.019 frequency of concordance ranging 0.015 to 0.025), tissue-specific mutations exhibited more fractions (median = 0.069 frequency of concordance ranging 0.037 to 0.013) (Fig. 1b). For instance, approximately 13% of total mutations in SNU-4026 and SNU-4954 series were tissue-specific, which was nearly twice as much as other sets. Continuous passaging of the cell lines functions as selective force to reduce heterogeneous cell populations, which might cause the decreased concordance rate between the original tumor and matched cell lines. We confirmed that the passages of all applied samples were set to 4–6, which excluded the potential de novo loss of mutations through the cell line establishment of SNU-4026 and SNU-4954. Other experimental settings were equivalent, and we concluded that this is due to random effect during the cell line establishment.

Mutational signatures of the tissue and cell lines were compared as well (Fig. 1c). The most predominant point mutation type in total samples was the C-to-T transition including CpG regions (Supplementary Fig. 1b) matching well to the other glioblastoma sequencing cohorts^10^. Since we applied different sequencing depths to the tissue and cell lines, we only compared the types of mutational signatures between tumor tissue and cell lines which was highly corresponding. The portion of signature 3 and 12 was decreased in most of the cell lines, which might imply the culture-derived selection favors specific mutational signatures.

**Fig. 2:**
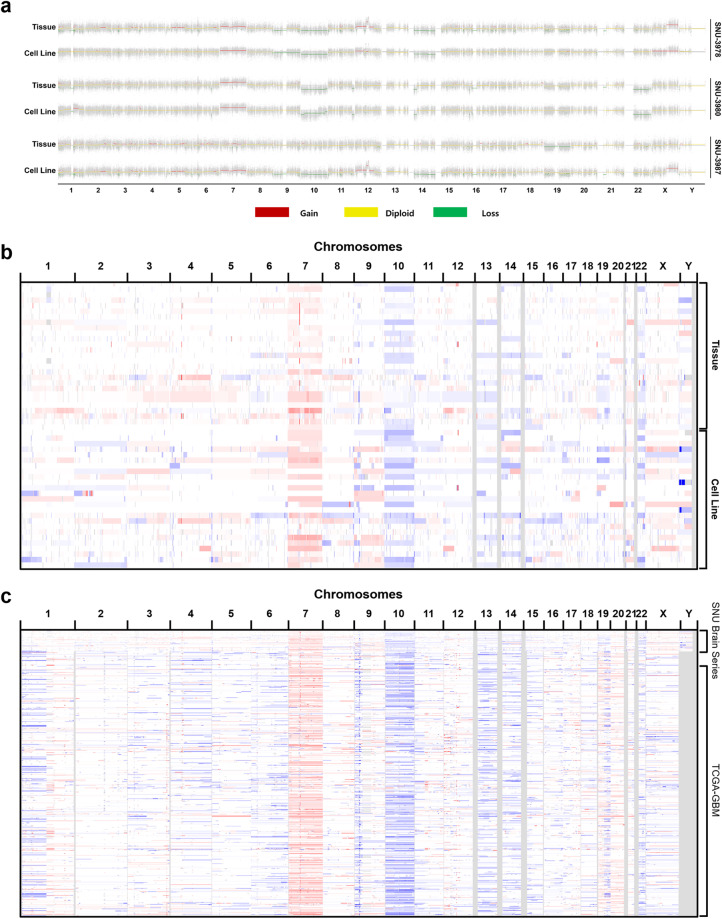
(**a**) Copy number analysis of exemplary three samples (SNU-3978, SNU-3980, and SNU-3987) shows that the cell lines reflect the copy number variation of its original tumors. (**b**) The overview of CNV indicates universal gain at chromosome 7 and loss at chromosome 10. (**c**) Comparison with TCGA-GBM cohort exhibited SNU GBM series were intermingled well with a larger cohort.

We also compared the exome-wide CNVs of cell lines to matched tumor tissues. Cell lines displayed mostly analogous CNV patterns with the parental tumors. Few changes in CNV were observed such as gain at chromosome 12p and loss at chromosome 14 of SNU-3978 sample (Fig. 2a). Our samples displayed comparable CNVs with a larger TCGA-GBM cohort^10^, which maintained the gain of chromosome 7 and loss of chromosome 10 (Fig. 2b,c). Inspection of the top regions identified by TCGA disclosed the presence of EGFR-amplified and CDKN2A, PTEN-depleted cell lines, as well as a documented gain of 19q region (Supplementary Fig. 2). Overall, this data validated that the GBM cancer cell lines recapitulate the genomic characteristics of the primary tumor and most of the genomic diversity of Glioblastoma.

We provide the aligned BAM files and the processed Variant Call format (VCF) files for each of the samples encompassing the variants of GATK HaplotypeCaller pipeline for variant genotyping for each sample based on the BAM file previously generated. These data can be a valuable resource for investigating genetic variants, genes and signaling pathways to identify novel factors related to these disorders, and may provide novel information for the investigation of the Korean population or in general for studies of genetic polymorphisms of human population.

## Method

### Establishment and maintenance of human GBM cell lines

This study was reviewed and approved by the institutional review board of the Seoul National University Hospital (IRB No. 1608-139-787), and written informed consent was obtained from all patients enrolled in this study. All data were handled anonymously. Surgical specimens and clinical information were obtained from 26 GBM patients who underwent surgery at Seoul National University Hospital. Informed consent was obtained from all patients for the usage of samples and the establishment of cell lines. Baseline patient and tumor information is summarized in Table 1. The histological diagnosis was rendered using the WHO 2016 classification. Cell lines of histologically proven GBM. Cell lines of histologically proven GBM were established. Solid tumors were finely minced with scissors and dispersed into small aggregates by pipetting. Appropriate amounts of fine neoplastic tissue fragments were seeded into 25 cm^2^ flasks. Most of the tumor cells were initially cultured in Opti-MEM medium supplemented with 5% heat-inactivated fetal bovine serum (FBS) (O5). Cultures were maintained in RPMI 1640 supplemented with 10% heat-inactivated FBS (R10). Initial passages were performed when heavy tumor cell growth was observed, and subsequent passages were performed every 1 or 2 weeks. Adherent cells were recovered while growth was subconfluent by treatment with trypsin, dispersed by pipetting and used for the passages. If stromal cell growth was noted in the initial cultures, differential trypsinization was used to obtain a pure tumor cell population. Cultures were maintained in humidified incubators at 37 °C in an atmosphere of 5% CO 2 and 95% air. All cell lines were confirmed to be free of mycoplasma contamination.

### DNA purification

Genomic DNA (gDNA) samples were isolated from the GBM tissue and blood DNA using the DNeasy Blood and Tissue Kit (Qiagen, MD, USA) according to the manufacturer’s recommendations for the spin-column protocol, using 30 mg starting tissue material from each sample. In short, tissue samples were cut into small pieces, and then lysis buffer (provided by the kit) and proteinase K were added. Lysis reactions were carried out at 56 °C until complete lysis was obtained. DNeasy Mini spin columns (kit’s component) were used for the isolation of gDNA from the lysate. Elution was carried out twice to a final volume of 100 μl per elution.

### DNA fingerprinting

DNA fingerprinting was proceeded with extracted gDNA. Quantified and diluted gDNA solution was added to reaction mixture consisted of Amp FISTR PCR reaction mix, Taq DNA polymerase, and Amp FISTR identifier primer set (Applied Biosystem, CA, USA). Then the sequence is amplified by GeneAmp PCR System 9700 (Applied Biosystem) with annealing temperature set to 59°C. 0.05 μl of Gene Scan-500 Rox standard and 9 μl of Hi-Di Formamide (Applied Biosystem) were added to 1 μl of PCR product of each cell line and denatured at 95°C for 2 minutes. This mixture was then analyzed by 3500xL Genetic Analyzer (Applied Biosystem).

### Sanger sequencing

For the hTERT promoter region sequencing, 1 μL of gDNA of each cell lines were amplified in 14 μL PCR mixture containing 1.5 μL of 10X PCR buffer with MgCl2, 0.5 μL of dNTP, 0.25 μl of forward primer, 0.25 μl of reverse primer, and 0.08 μL of Taq DNA polymerase (Intron Biotechnology, Kyung-gi, South Korea) was proceeded using GeneAmp PCR System 9700 (Applied Biosystems, CA, USA). Each PCR cycle was set with denaturation step at 96°C annealing temperature at 68°C, and elongation at 72°C for 35 cycles. The primer sequence is following: hTERT-F > CTGGCGTCCCTGCACCCTGG, hTERT-R > ACGAACGTGGCCAGCGGCAG with estimated amplicon size of 470 bp. PCR product was precipitated by 5% sodium acetate buffer (Sigma-Aldrich, Cat# S7899) and 95% ethanol mixed solution. Then washed product was set on ice for 10 minutes and centrifuged at 4°C, 14000 rpm. Supernatant was discarded and the product was rinsed this time by 70% ethanol and centrifuged 14000 rpm. Supernatant was discarded then the products were dried using vacuum concentrator (Eppendorf). 10 μL of distilled water was added to dilute precipitated sample. When the product is all diluted in distilled water, cyclic PCR was carried out. Two separate mixtures for forward and reverse sequences were made where they each include 5X sequencing buffer (Applied Biosystems), Big Dye (Applied Biosystems), forward or reverse primer, distilled water, and product from the previous step. Cyclic PCR was carried out with denaturation step at 96°C, annealing temperature at 55°C, and elongation at 60°C for 25 cycles. The cyclic PCR product was then precipitated with 5% sodium acetate buffer and 95% ethanol mixed solution and set on ice for 10 minutes then it was centrifuged at 4°C and supernatants were carefully discarded and the final product was dried using the vacuum concentrator. 10 μL Hi-Di formamide (Applied Biosystems) was added to dilute the dried product. This final product was transferred to 96 well PCR plate and denatured at 95°C for 2 minutes before taken to 3500xL Genetic Analyzer (Applied Biosystems) for sequencing.

### Quality and quantity check of DNA

The generation of standard exome capture libraries, we used the Agilent SureSelect Target Enrichment protocol for Illumina paired-end sequencing library (ver. B.3, June 2015) together with 200 ng input gDNA. In all cases, the SureSelect Human All Exon V6 probe set was used. The quantification of DNA and the DNA quality is measured by PicoGreen and Nanodrop. Fragmentation of 1ug of genomic DNA was performed using adaptive focused acoustic technology. (AFA; Covaris) The fragmented DNA is repaired, an ‘A’ is ligated to the 3′ end, agilnet adapters are then ligated to the fragments. Once ligation had been assessed, the adapter ligated product is PCR amplified. The final purified product is then quantified using qPCR according to the qPCR Quantification Protocol Guide and qualified using the Caliper LabChipHigh Sensitivity DNA. (PerkinElmer). For exome capture, 250 ng of DNA library was mixed with hybridization buffers, blocking mixes, RNase block and 5 µl of SureSelect all exon capture library, according to the standard Agilent SureSelect Target Enrichment protocol. Hybridization to the capture baits was conducted at 65 °C using heated thermal cycler lid option at 105 °C for 24 hours on PCR machine. The captured DNA was then amplified. The final purified product is then quantified using qPCR according to the qPCR Quantification Protocol Guide and qualified using the TapeStationDNAscreentape(Agilent). And then we sequenced using the HiSeq™ 2500 platform (Illumina, San Diego, USA).

### Whole-exome sequencing

Whole-exome capture was performed on all samples with the SureSelect Human All Exon V5 Kit (Agilent Technologies, Tokyo, Japan). The captured targets were subjected to sequencing using HiSeq. 2500 (Illumina, San Diego, CA, USA) with the pair-end 100 bp read option for cell lines and blood samples and 200 bp read option for tissue materials. Information on read depth is provided in Supplementary Data 2. The sequence data were processed through an in-house pipeline. Briefly, paired-end sequences were firstly mapped to the human genome, where the reference sequence was UCSC assembly hg19 (original GRCh37 from NCBI, Feb. 2009) using the mapping program BWA (version 0.7.12), and generated a mapping result file in BAM format using BWA-MEM. Then, Picard-tools (ver.1.130) were applied in order to remove PCR duplicates. The local realignment process was performed to locally realign reads with BAM files reducing those reads identically match to a position at start into a single one, using MarkDuplicates.jar, which required reads to be sorted. By using Genome Analysis Toolkit, base quality score recalibration (BQSR) and local realignment around indels were performed. Haplotype Caller of GATK (GATKv3.4.0) was used for variant genotyping for each sample based on the BAM file previously generated (SNP and short indels candidates are detected). Somatic mutations were identified by providing the reference and sequence alignment data of tumor tissues or cell lines to the MuTect2 (involved in GATK v3.8.0) with default parameters using tumor-normal mode. Those variants were annotated by SnpEff v4.1 g, to vcf file format, filtering with dbSNP for the version of 142 and SNPs from the 1000 genome project. Then, SnpEff was applied to filter additional databases, including ESP6500, ClinVar, dbNSFP 2.9. Mutational signatures were evaluated using the Mutational Patterns R package, release 3.6.1 to configure distinct footprints in genomic context for all somatic SNVs and evaluate a multitude of mutational patterns in base substitution in tumor tissues and matched cell lines.

**Fig. 3:**
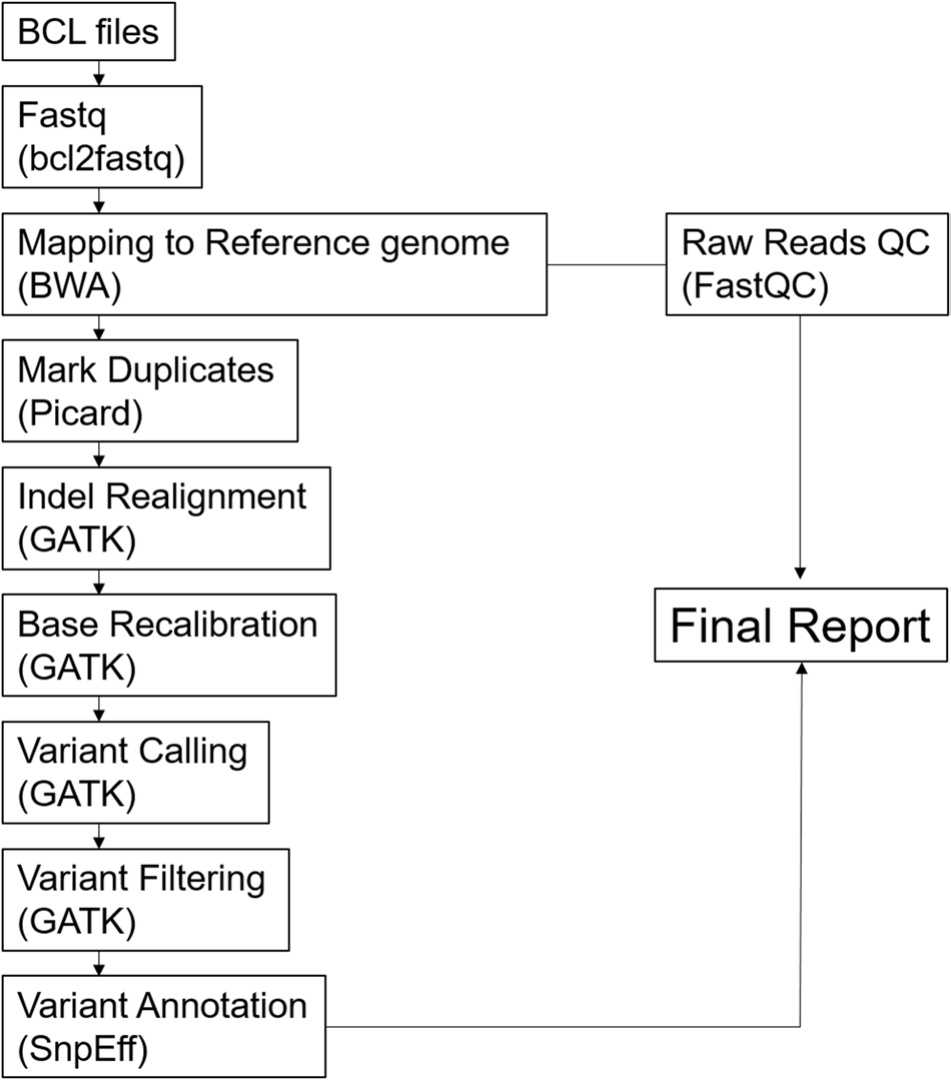
Data flow diagram shows the detailed overview of the analysis design and bioinformatics pipelines.

**Fig. 4:**
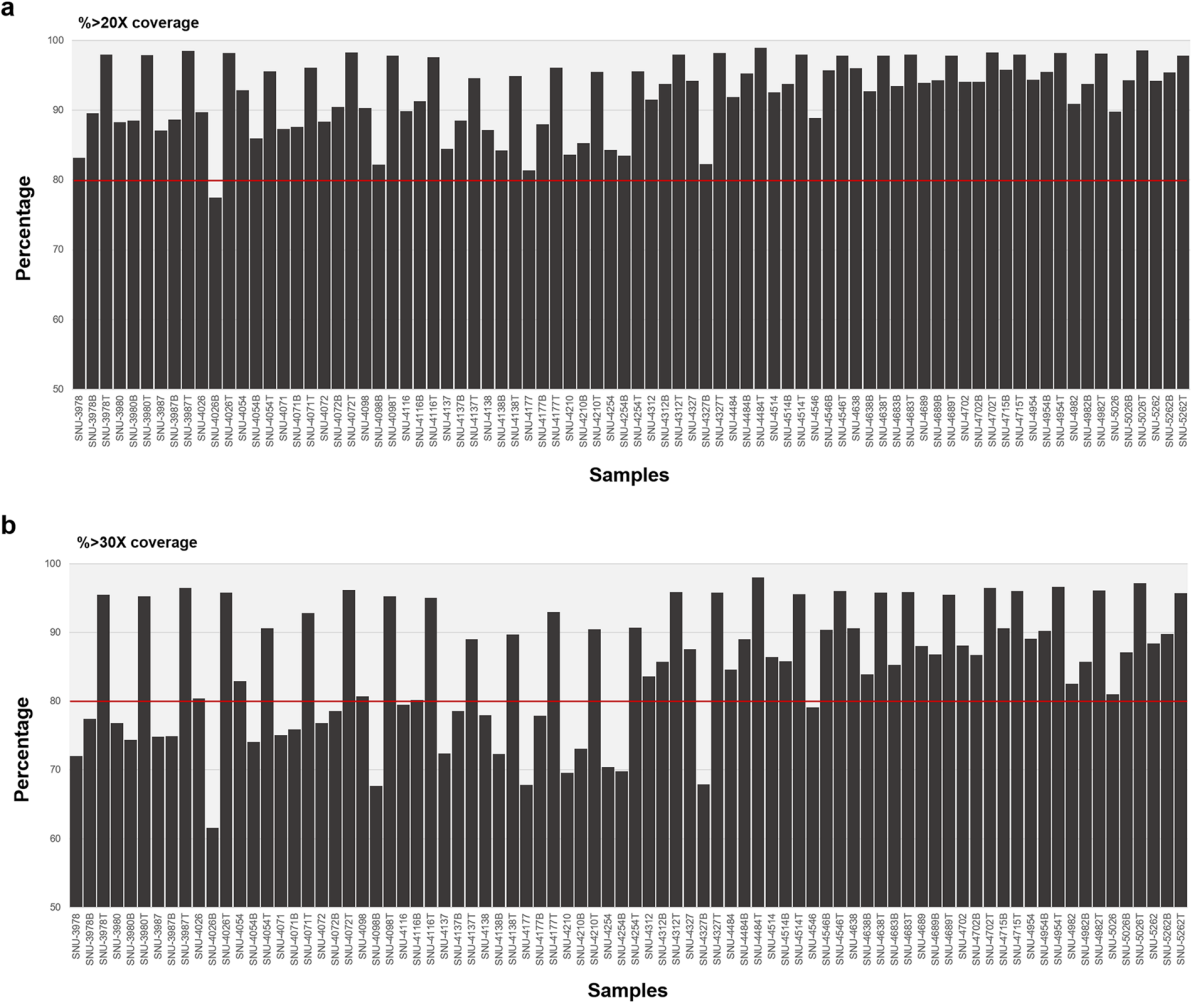
WES coverage for the at 20X and 30X depth. Panels show coverage at 20X (**a**) and 30X (**b**). Each bar represents an individual sample and the percentage of bases with at least 20X or 30X coverage. Red lines mark 80% coverage at 20X and 30X depths, respectively.

We performed high-depth, short-read, and paired-end WES on fresh-frozen collection of GBM tissue and matched blood samples from 26 GBM patients. Here we describe the sample collection methods, the library preparation and sequencing method, the currently available data records, and technical validations for our dataset. A schematic overview of this study, including the bioinformatics workflow, is also presented (Fig. 3). The DNA samples were sequenced using SureSelect Human All Exon V5 Kit. The captured targets were subjected to sequencing using HiSeq. 2500 with the pair-end 100 bp read option for cell lines and blood samples and 200 bp read option for tissue materials in order to counterbalance dissimilar cellularity of tumor cells between tumor tissues and cell lines, which resulted in an average of 167 million paired-end reads for tissues samples and 83.5 million paired-end reads for blood and cell lines samples. Reads were aligned to the hg19 reference human genome, and we obtained high coverage per base position in both tumor tissue and cell lines. In average, we determined 94,044 single nucleotide polymorphisms (SNPs) and 12,518 insertions/deletions (indels) per sample. Variants presented in this cohort were found of 96.1% in dbSNP v142. An average transition/transversion (Ts/Tv) ratio was 2.26 (Table 2). In the tumor tissue cohort 88.6–99.0% (quartiles) of target regions had higher than 20-fold and 77.9–98.0% of target regions had higher than 30-fold coverage. In the blood and cell line cohort, these values were 87.2–96% for 20-fold and 74.7–90.6% for 30-fold coverage, respectively (Fig. 4, Table 3). The information of post-alignment is summarized in Table 4.

**Table 2:** Number and types of mutation found in cell line, blood and tissue of 26 glioblastoma patients.

SNU Number	# of SNP	Synonymous Variant	Missense Variant	Stop Gained	Stop Lost	# of INDEL	Frameshift Variant	Inframe Insertion	Inframe Deletion	% Found in dbSNP142	Het/Hom Ratio	Ts/Tv Ratio
SNU-3978	87259	11574	11016	116	36	9737	304	163	178	96.4	1.1	2.3
SNU-3978B	94615	11931	11289	111	37	10833	302	163	189	96.7	1.3	2.3
SNU-3978T	99363	12076	11546	120	37	13409	327	177	196	95.9	1.4	2.2
SNU-3980	89607	11377	10861	103	38	10574	307	167	171	96.7	1	2.3
SNU-3980B	93095	11714	11234	110	36	10826	310	171	175	96.8	1.3	2.3
SNU-3980T	97587	11842	11373	108	36	13110	329	180	178	95.9	1.3	2.2
SNU-3987	88748	11336	11036	111	37	9931	327	164	184	96.7	1.1	2.3
SNU-3987B	93295	11723	11384	108	42	10927	346	169	185	96.7	1.3	2.3
SNU-3987T	98823	11900	11536	113	40	13619	358	179	201	95.7	1.4	2.2
SNU-4026	83223	10637	10222	88	38	9804	294	149	186	96.7	0.7	2.3
SNU-4026B	89783	11812	11239	95	42	10028	315	170	210	96.6	1.2	2.3
SNU-4026T	98348	12017	11508	100	42	13418	340	173	210	95.8	1.4	2.2
SNU-4054	96182	11994	11388	107	39	13423	303	172	200	96.3	1.3	2.3
SNU-4054B	91632	11946	11274	109	41	10279	297	159	198	97	1.3	2.3
SNU-4054T	98228	12130	11544	120	40	12514	339	177	205	95.8	1.4	2.3
SNU-4071	88803	11441	11138	107	40	11318	315	168	183	96.7	1	2.3
SNU-4071B	94379	11841	11511	108	41	11362	311	167	194	96.5	1.3	2.3
SNU-4071T	97041	12009	11679	113	42	12390	321	186	209	96.1	1.3	2.2
SNU-4072	90987	11455	10878	108	38	10334	295	165	183	96.7	1.1	2.3
SNU-4072B	94149	11808	11188	109	40	10957	310	177	193	96.8	1.3	2.3
SNU-4072T	99682	11986	11490	116	41	13442	321	187	203	95.8	1.4	2.2
SNU-4098	90137	11506	11011	106	39	10534	312	163	187	96.5	1.1	2.3
SNU-4098B	90961	11837	11382	107	41	10345	331	176	199	96.7	1.3	2.3
SNU-4098T	98202	12008	11591	107	41	13440	347	182	206	95.8	1.4	2.2
SNU-4116	90850	11483	11095	100	39	10617	297	182	194	96.7	1.1	2.3
SNU-4116B	94290	11862	11393	109	42	11141	308	182	198	96.8	1.3	2.3
SNU-4116T	98438	11975	11620	109	40	13132	313	191	212	96.3	1.4	2.3
SNU-4137	88893	11565	11045	127	43	9992	315	165	196	96.3	1.1	2.3
SNU-4137B	92922	11913	11332	128	39	10785	314	176	203	96.6	1.3	2.3
SNU-4137T	97163	11969	11494	132	42	12129	339	176	205	95.9	1.3	2.2
SNU-4138	88236	11488	10970	134	38	10162	302	169	191	96.6	1.1	2.3
SNU-4138B	88711	11878	11291	124	40	9876	310	170	201	96.8	1.3	2.3
SNU-4138T	98436	12073	11636	133	41	12296	342	187	214	96.1	1.4	2.3
SNU-4177	82232	10966	10433	91	34	9086	298	146	180	96.7	0.8	2.3
SNU-4177B	92278	11875	11250	96	37	10507	316	166	200	96.8	1.3	2.3
SNU-4177T	96410	11957	11388	99	38	12416	336	171	205	96.1	1.3	2.3
SNU-4210	89754	11571	11078	114	42	9831	312	168	199	96.6	1.1	2.3
SNU-4210B	89977	11787	11165	109	37	9972	310	163	206	96.8	1.2	2.3
SNU-4210T	97669	11975	11504	113	38	12553	331	179	214	96.1	1.3	2.2
SNU-4254	90340	11750	11095	110	37	9923	331	162	198	96.8	1.2	2.3
SNU-4254B	89656	11732	11094	118	37	9951	328	161	196	96.8	1.2	2.3
SNU-4254T	97165	11920	11352	120	41	12261	347	177	214	95.9	1.3	2.2
SNU-4312	87683	11091	10834	110	37	10634	294	150	166	96.6	0.9	2.3
SNU-4312B	95829	11908	11514	113	42	11803	331	168	181	96.5	1.3	2.3
SNU-4312T	96446	11731	11452	124	44	13196	338	174	188	95.7	1.3	2.3
SNU-4327	92672	11659	11029	112	39	11411	303	172	195	96.3	1.1	2.3
SNU-4327B	90828	11834	11216	112	43	10188	282	179	190	96.7	1.2	2.3
SNU-4327T	98565	12026	11477	116	46	13313	318	186	198	95.7	1.4	2.2
SNU-4484	89241	11214	10637	102	35	12548	321	150	191	96.3	0.9	2.3
SNU-4484B	97245	12050	11424	111	37	14123	337	178	212	95.9	1.3	2.2
SNU-4484T	100071	12108	11590	118	35	16466	350	176	208	94.9	1.4	2.2
SNU-4514	89817	11375	10816	112	38	12038	316	160	189	96	1	2.3
SNU-4514B	97811	12122	11622	112	41	14103	350	182	214	96.1	1.4	2.3
SNU-4514T	96118	11736	11295	117	40	15176	342	185	206	95.3	1.2	2.2
SNU-4546	90736	11482	10922	105	36	11892	297	171	179	96.4	1.1	2.3
SNU-4546B	97644	11965	11435	104	38	14526	324	190	191	95.8	1.4	2.3
SNU-4546T	97979	11917	11416	112	39	15431	326	194	178	95.3	1.4	2.2
SNU-4638	98532	11989	11481	98	43	15045	315	175	191	96.1	1.4	2.3
SNU-4638B	97367	11947	11482	103	42	13841	318	177	195	96.2	1.4	2.3
SNU-4638T	99877	12013	11612	106	38	15734	329	178	198	95.6	1.5	2.2
SNU-4689	91987	11534	10976	119	41	13031	322	162	193	96	1	2.3
SNU-4689B	97298	12026	11494	128	42	14061	356	178	216	96	1.3	2.2
SNU-4689T	98152	12019	11554	133	42	15378	356	173	220	95.2	1.4	2.2
SNU-4702	92163	11423	11041	97	33	13091	313	160	185	96	1.1	2.3
SNU-4702B	96775	11871	11442	103	38	13786	343	178	192	96.2	1.3	2.2
SNU-4702T	99216	11946	11615	109	34	15665	346	177	199	95.3	1.4	2.2
SNU-4954	75108	9237	8878	85	38	11225	272	147	161	95.5	0.2	2.3
SNU-4954B	98611	12112	11538	114	42	14380	344	188	207	95.9	1.4	2.3
SNU-4954T	85662	10503	10134	99	40	14131	309	162	180	94.7	0.7	2.2
SNU-4982	91731	11528	11076	105	36	12433	323	161	205	96.3	1.1	2.3
SNU-4982B	96517	11893	11428	109	36	14023	349	169	224	96	1.3	2.2
SNU-4982T	98703	11985	11594	107	36	15623	358	171	224	95.3	1.4	2.2
SNU-5026	90272	11364	10894	104	43	12302	300	146	167	96.4	1	2.3
SNU-5026B	96492	11903	11426	111	44	13856	324	158	185	96.2	1.4	2.2
SNU-5026T	99425	12028	11665	123	44	16016	331	168	195	95.2	1.4	2.2
SNU-5262	92714	11491	10998	105	37	13313	321	161	179	96	1.1	2.3
SNU-5262B	97579	11968	11421	105	37	14438	349	170	188	96.2	1.4	2.2
SNU-5262T	99191	12075	11602	107	38	15669	357	170	194	95.5	1.4	2.2

**Table 3:** Percent coverage of target region.

SNU Number	% >20X	SNU Nubmer	% >30X
SNU-3978	83.2	SNU-3978	72
SNU-3978B	89.6	SNU-3978B	77.4
SNU-3978T	98	SNU-3978T	95.5
SNU-3980	88.3	SNU-3980	76.8
SNU-3980B	88.5	SNU-3980B	74.4
SNU-3980T	97.9	SNU-3980T	95.3
SNU-3987	87.1	SNU-3987	74.8
SNU-3987B	88.7	SNU-3987B	74.9
SNU-3987T	98.5	SNU-3987T	96.5
SNU-4026	89.7	SNU-4026	80.4
SNU-4026B	77.5	SNU-4026B	61.6
SNU-4026T	98.2	SNU-4026T	95.8
SNU-4054	92.9	SNU-4054	82.9
SNU-4054B	86	SNU-4054B	74.1
SNU-4054T	95.6	SNU-4054T	90.6
SNU-4071	87.3	SNU-4071	75.1
SNU-4071B	87.6	SNU-4071B	75.9
SNU-4071T	96.1	SNU-4071T	92.8
SNU-4072	88.4	SNU-4072	76.8
SNU-4072B	90.5	SNU-4072B	78.6
SNU-4072T	98.3	SNU-4072T	96.2
SNU-4098	90.3	SNU-4098	80.7
SNU-4098B	82.2	SNU-4098B	67.7
SNU-4098T	97.8	SNU-4098T	95.3
SNU-4116	89.9	SNU-4116	79.5
SNU-4116B	91.3	SNU-4116B	80.2
SNU-4116T	97.6	SNU-4116T	95
SNU-4137	84.5	SNU-4137	72.4
SNU-4137B	88.5	SNU-4137B	78.6
SNU-4137T	94.6	SNU-4137T	89
SNU-4138	87.2	SNU-4138	78
SNU-4138B	84.2	SNU-4138B	72.3
SNU-4138T	94.9	SNU-4138T	89.7
SNU-4177	81.4	SNU-4177	67.8
SNU-4177B	88	SNU-4177B	77.9
SNU-4177T	96.1	SNU-4177T	93
SNU-4210	83.6	SNU-4210	69.6
SNU-4210B	85.3	SNU-4210B	73.1
SNU-4210T	95.5	SNU-4210T	90.5
SNU-4254	84.3	SNU-4254	70.4
SNU-4254B	83.5	SNU-4254B	69.8
SNU-4254T	95.6	SNU-4254T	90.7
SNU-4312	91.5	SNU-4312	83.6
SNU-4312B	93.8	SNU-4312B	85.7
SNU-4312T	98	SNU-4312T	95.9
SNU-4327	94.2	SNU-4327	87.6
SNU-4327B	82.3	SNU-4327B	67.9
SNU-4327T	98.2	SNU-4327T	95.8
SNU-4484	91.9	SNU-4484	84.6
SNU-4484B	95.3	SNU-4484B	89
SNU-4484T	99	SNU-4484T	98
SNU-4514	92.6	SNU-4514	86.4
SNU-4514B	93.8	SNU-4514B	85.8
SNU-4514T	98	SNU-4514T	95.6
SNU-4546	88.9	SNU-4546	79.1
SNU-4546B	95.7	SNU-4546B	90.4
SNU-4546T	97.8	SNU-4546T	96
SNU-4638	96	SNU-4638	90.6
SNU-4638B	92.7	SNU-4638B	83.9
SNU-4638T	97.8	SNU-4638T	95.8
SNU-4689	93.9	SNU-4689	88
SNU-4689B	94.3	SNU-4689B	86.8
SNU-4689T	97.8	SNU-4689T	95.5
SNU-4702	94.1	SNU-4702	88.1
SNU-4702B	94.1	SNU-4702B	86.7
SNU-4702T	98.3	SNU-4702T	96.5
SNU-4954	94.4	SNU-4954	89.1
SNU-4954B	95.5	SNU-4954B	90.2
SNU-4954T	98.2	SNU-4954T	96.6
SNU-4982	90.9	SNU-4982	82.5
SNU-4982B	93.8	SNU-4982B	85.7
SNU-4982T	98.1	SNU-4982T	96.1
SNU-5026	89.8	SNU-5026	81
SNU-5026B	94.3	SNU-5026B	87.1
SNU-5026T	98.6	SNU-5026T	97.2
SNU-5262	94.2	SNU-5262	88.4
SNU-5262B	95.4	SNU-5262B	89.8
SNU-5262T	97.8	SNU-5262T	95.7
Min	77.5	Min	61.6
Max	99	Max	98
Average	92.24615385	Average	84.77307692
Q1	88.5	Q1	77.525
Q2	93.85	Q2	86.55
Q3	96.1	Q3	92.95
Q4	99	Q4	98

**Table 4:** Post-alignment reads information.

SNU Number	Initial Mappable Reads	% Initial Mappable Reads	Non-Redundant Reads	% Non-Redundant Reads	On-Target Reads	% On-Target Reads	On-Target Yield (bp)	Mean Depth of Target Regions
SNU-3978	70385168	99.8	62820416	89.2	50392448	80.2	4416719903	73
SNU-3978B	67849353	99.8	63755919	93.9	44333517	69.5	3840173736	63.5
SNU-3978T	136545691	99.8	121760894	89.1	88273737	72.4	7614427475	125.9
SNU-3980	67480745	99.8	61676930	91.3	47056060	76.2	4128053610	68.2
SNU-3980B	60768632	99.8	56348971	92.7	38339615	68	3319161266	54.9
SNU-3980T	141700631	99.8	126933448	89.5	91330539	71.9	7883092682	130.3
SNU-3987	59331697	99.8	55347380	93.2	41407621	74.8	3603855976	59.6
SNU-3987B	64227395	99.8	59614702	92.8	40259602	67.5	3483417248	57.6
SNU-3987T	153882576	99.8	136414028	88.6	97746794	71.6	8410734806	139.1
SNU-4026	73062414	99.8	67887106	92.9	51781289	76.2	4528302022	74.9
SNU-4026B	61764039	99.8	56498403	91.4	41064328	72.6	3565789323	58.9
SNU-4026T	144596209	99.8	128959694	89.1	94083366	72.9	8108960105	134.1
SNU-4054	75476554	99.8	68574838	90.8	47809048	69.7	4123278057	68.2
SNU-4054B	67298298	99.6	62446582	92.7	45907289	73.5	3973381393	65.7
SNU-4054T	139526274	99.8	125936711	90.2	85064419	67.5	7321360632	121.1
SNU-4071	60367532	99.8	54444507	90.1	42185508	77.4	3670548043	60.7
SNU-4071B	74433999	99.8	63538539	85.3	42704360	67.2	3623243102	59.9
SNU-4071T	164917878	99.8	149692315	90.7	110625851	73.9	9625579977	159.2
SNU-4072	62706529	99.8	58441402	93.1	42755930	73.1	3708358985	61.3
SNU-4072B	65760997	99.8	61615575	93.6	42191123	68.4	3656058047	60.4
SNU-4072T	152151850	99.8	135941258	89.3	97769549	71.9	8424832423	139.3
SNU-4098	72121550	99.8	67208349	93.1	49013892	72.9	4256685094	70.4
SNU-4098B	71143164	99.8	63839175	89.7	46071544	72.1	3998457256	66.1
SNU-4098T	134236266	99.8	120803561	89.9	87275798	72.2	7519839156	124.3
SNU-4116	68302204	99.8	63914408	93.5	45575097	71.3	3939204748	65.1
SNU-4116B	66373411	99.8	62231872	93.7	42393689	68.1	3657794168	60.5
SNU-4116T	130900987	99.8	119022950	90.9	85325813	71.6	7348727301	121.5
SNU-4137	68147124	99.5	63021901	92.4	47800146	75.8	4135232042	68.4
SNU-4137B	75401073	99.2	70113134	92.9	51876690	73.9	4461844255	73.8
SNU-4137T	130351347	99.9	117491762	90.1	78109102	66.4	6720427619	111.1
SNU-4138	76311720	99.3	69864723	91.5	53618045	76.7	4661286209	77.1
SNU-4138B	61031390	99.9	55239434	90.5	42215451	76.4	3703695641	61.2
SNU-4138T	129032468	99.8	116457464	90.2	77819981	66.8	6690754659	110.6
SNU-4177	59464035	99.9	54395319	91.4	41292229	75.9	3612956995	59.7
SNU-4177B	73744590	99.1	67806809	91.9	52028536	76.7	4484794839	74.1
SNU-4177T	173175019	99.8	157801625	91.1	115377323	73.1	10034055583	165.9
SNU-4210	72472360	99.8	65705011	90.6	44624564	67.9	3838791645	63.5
SNU-4210B	65365044	99.6	59822979	91.5	44413506	74.2	3850811215	63.7
SNU-4210T	139652925	99.8	126831638	90.8	83458913	65.8	7173041362	118.6
SNU-4254	64940347	99.9	59019469	90.8	43404486	73.5	3754117530	62.1
SNU-4254B	62723481	99.8	57424530	91.5	41827992	72.8	3631040240	60
SNU-4254T	140032839	99.8	123568079	88.2	82764522	66.9	7111262514	117.6
SNU-4312	92890277	99.8	83720241	90.1	60826471	72.6	5339071038	88.3
SNU-4312B	79231288	99.8	73142999	92.3	51685217	70.6	4467887301	73.9
SNU-4312T	160402552	99.9	143263918	89.3	103478066	72.2	8929018818	147.6
SNU-4327	92171965	99.8	83414866	90.4	59226125	71	5126213545	84.7
SNU-4327B	66721310	99.8	59616088	89.3	43820907	73.5	3805503406	62.9
SNU-4327T	144168438	99.8	128495489	89.1	92510771	71.9	7977557100	131.9
SNU-4484	97285377	99.8	87438291	89.8	67296065	76.9	5870267985	97.1
SNU-4484B	122814912	99.8	109843710	89.4	72288536	65.8	6231728355	103
SNU-4484T	298108251	99.7	267235691	89.6	174198814	65.1	14943518431	247.1
SNU-4514	100135447	99.8	89728211	89.6	67192716	74.8	5844049252	96.6
SNU-4514B	100337810	99.8	92249156	91.9	61024434	66.1	5232778621	86.5
SNU-4514T	182696464	99.8	166611339	91.1	105600910	63.3	9032701563	149.4
SNU-4546	80397632	99.8	72610608	90.3	50744448	69.8	4411657509	72.9
SNU-4546B	128527863	99.8	117317924	91.2	76768714	65.4	6579489888	108.8
SNU-4546T	175192215	99.8	160182826	91.4	112336612	70.1	9679703296	160.1
SNU-4638	103514313	99.8	86872492	83.9	66117497	76.1	5731135582	94.8
SNU-4638B	103913895	99.8	95754370	92.1	58651792	61.2	5031209139	83.2
SNU-4638T	161508356	99.8	149772666	92.7	102165788	68.2	8761131087	144.9
SNU-4689	108883134	99.8	98112354	90.1	72193676	73.5	6248070015	103.3
SNU-4689B	105934526	99.8	97307498	91.8	61876189	63.5	5288486566	87.4
SNU-4689T	178410387	99.8	162371884	91	105978816	65.2	9072282905	150
SNU-4702	99010373	99.8	90970729	91.8	65210586	71.6	5616512171	92.9
SNU-4702B	98302951	99.8	90393722	91.9	59347019	65.6	5077228718	83.9
SNU-4702T	190292163	99.8	172847959	90.8	112372139	65	9629224556	159.2
SNU-4954	115608424	99.8	103378247	89.4	74550170	72.1	6467502324	106.9
SNU-4954B	118444221	99.8	108301167	91.4	73872030	68.2	6349043829	105
SNU-4954T	230460838	99.8	207045614	89.8	130616195	63	11166182539	184.7
SNU-4982	82929671	99.8	75394682	90.9	55716629	73.8	4818084424	79.6
SNU-4982B	101180674	99.8	94073437	92.9	63032072	67	5429474133	89.8
SNU-4982T	186722136	99.8	170818931	91.4	107348738	62.8	9183425309	151.9
SNU-5026	81301607	99.8	74069771	91.1	55960470	75.5	4862212530	80.4
SNU-5026B	101032047	99.8	92845058	91.8	63484323	68.3	5465209384	90.4
SNU-5026T	221105706	99.8	199836309	90.3	129081712	64.5	11050849790	182.7
SNU-5262	105604721	99.8	94658206	89.6	65443595	69.1	5620894740	92.9
SNU-5262B	105200894	99.8	96383035	91.6	63302794	65.6	5416169992	89.5
SNU-5262T	172738249	99.8	157228640	91	100746439	64	8627974424	142.7

### Analysis of CNVs

For the detection of Copy Number Variations (CNVs) and loss of heterozygosity (LOH) from exome sequencing data, we employed ExomeCNV package in R program. The final log ratio of depth of coverage was determined by the number of bases targeted by exome sequencing (targeted base) and the number of bases actually sequenced (mapped). CNV calls were expressed as 1, 2, and 3 which indicated deletion, normal and amplification respectively.

## Data Records

The raw FASTQ files are deposited in the Sequence Read Archive (SRA) governed by National Center for Biotechnology Information (NCBI) with accession number (PRJNA896722)^11^. The GRCh37 aligned BAM files are deposited in the SRA with accession number (PRJNA896722). Data are publicly available at https://www.ncbi.nlm.nih.gov/bioproject/PRJNA896722. All cell lines introduced in this study including its genomic characterization will be deposited to Korean Cell Line Bank (http://cellbank.snu.ac.kr) at initial passages to be distributed to researchers worldwide.

## Technical Validation

### Quantitation of the purified DNA samples

The isolated DNA samples were quantified by PicoGreen and Nanodrop. DNA samples were diluted to 4 ng/μl with 1X Low TE Buffer. NanoDrop (Thermo Fischer Scientific) measurements were also performed to assess quantity and quality of DNA, 260:280 and 260:230 ratios greater to 1.8 were accepted.

### Quality control of the sheared DNA samples

The quality of the sheared DNA samples (200 ng of each) were checked prior to downstream analysis, using the Agilent Bioanalyser 2100 (Agilent Technologies), and High Sensitivity DNA chip and reagent kit. The electropherogram showed a DNA fragment size peak (for each of the samples) at around 150 bps.

### Quality check of the amplified samples

Agilent Bioanalyser 2100 (Agilent Technologies) and DNA 1000 Assay were used for the quality and quantity control of the libraries after PCR. The sample fragments sizes were between 250 and 275 bps.

### Quality control of raw reads and sample statistics

**Fig. 5:**
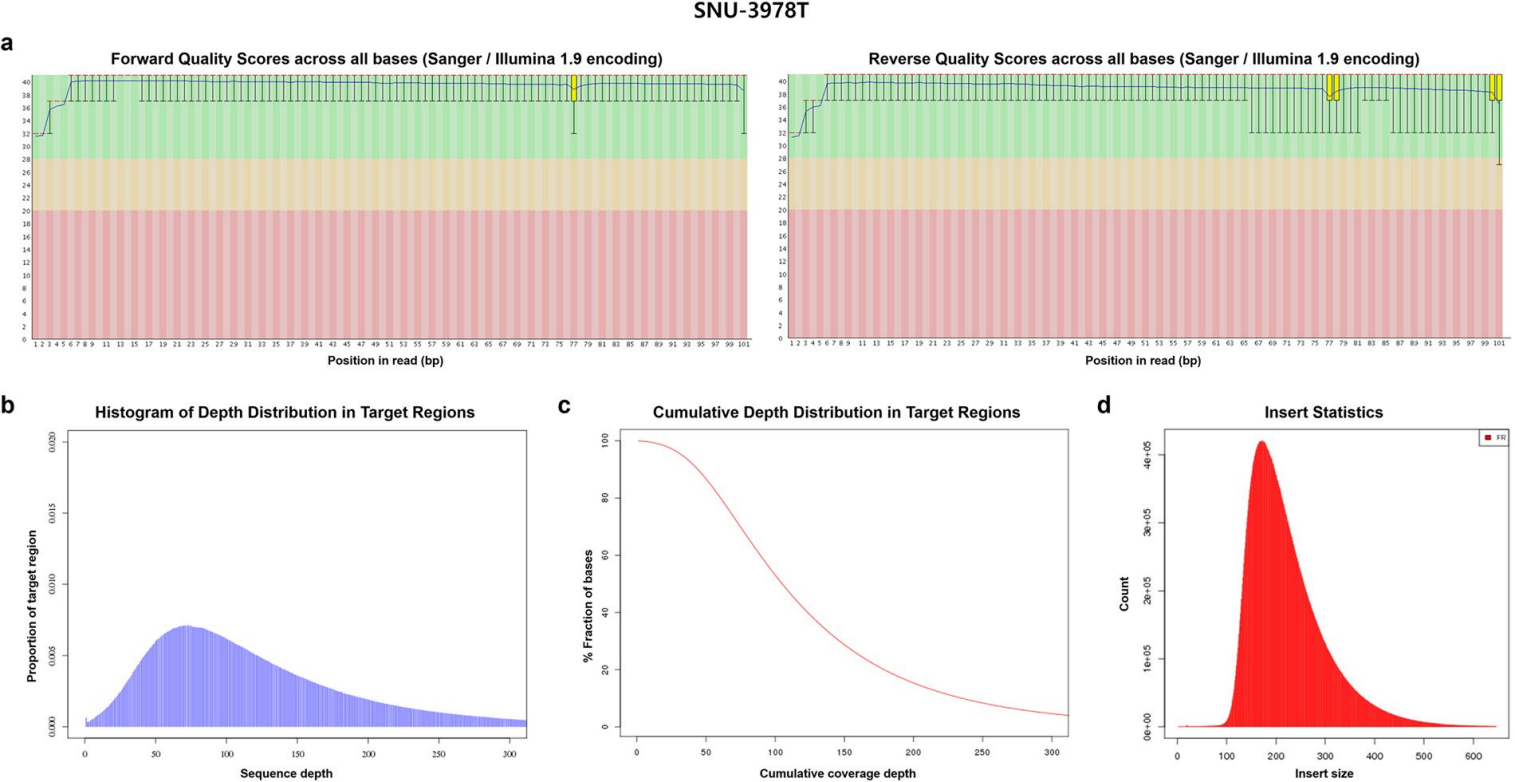
The sequence reads quality of SNU-3978T as a representative sample of tissue sample was summarized. The forward and reverse quality scores across all bases, depth distribution in target regions, cumulative depth distribution in target regions, and insert size of exemplary tissue sample are described.

**Fig. 6:**
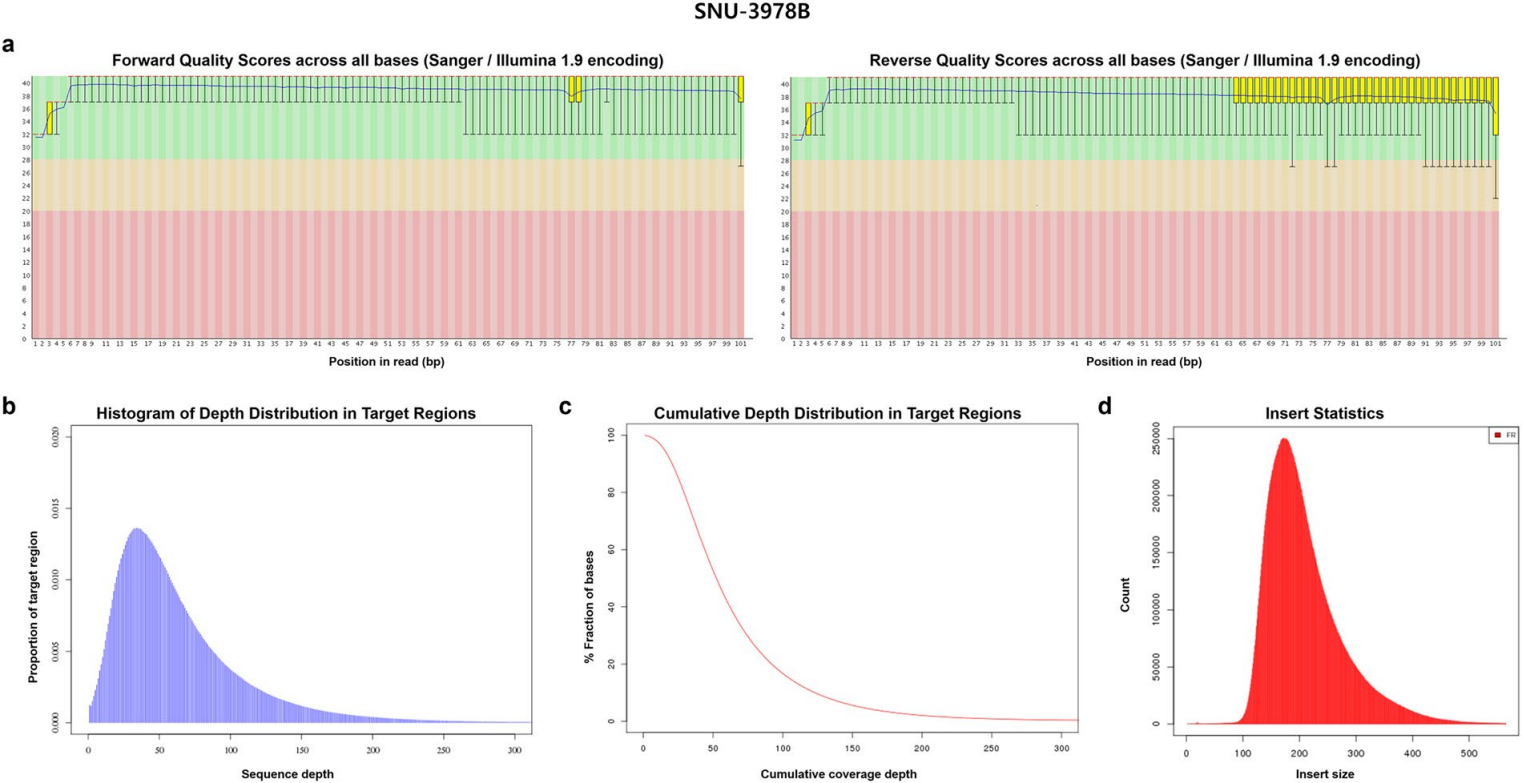
The sequence reads quality of SNU-3978B as a representative sample of tissue sample was summarized. The forward and reverse quality scores across all bases, depth distribution in target regions, cumulative depth distribution in target regions, and insert size of exemplary blood sample are described.

**Fig. 7:**
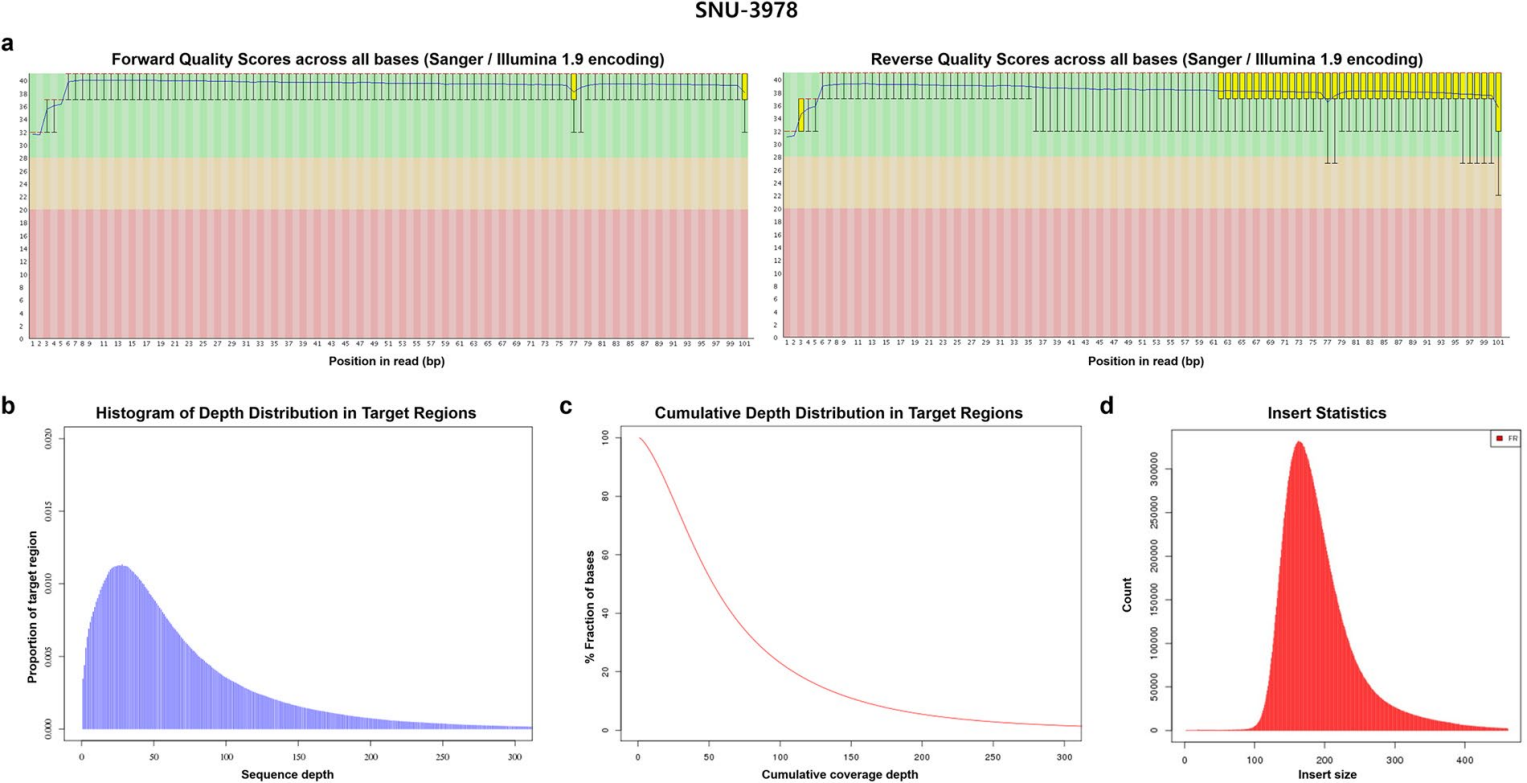
The sequence reads quality of SNU-3978 as a representative sample of tissue sample was summarized. The forward and reverse quality scores across all bases, depth distribution in target regions, cumulative depth distribution in target regions, and insert size of exemplary cell line sample are described.

Illumina BCL files were converted to FASTQ files by the standard Illumina protocol to remove low-quality reads and adaptors. The forward and reverse quality scores across all bases, depth distribution in target regions, cumulative depth distribution in target regions, and insert size are summarized in Figs. 5–7 for tissue, blood and cell lines samples respectively. Information on mappable reads and on-target reads are summarized in Table 4.

## Supplementary information

Supplementary Information

Dataset 1

Dataset 2

## Data Availability

Computational pipelines in this study are available at the public repository (https://github.com/mario2437/BrainTumor_ScientificData/blob/main/Code_Availability).
