# Author Correction: Patient-derived glioblastoma cell lines with conserved genome profiles of the original tissue

**DOI:** 10.1038/s41597-023-02390-x

**Published:** 2023-07-20

**Authors:** Soon-Chan Kim, Young-Eun Cho, Young-Kyoung Shin, Hyeon Jong Yu, Tamrin Chowdhury, Sojin Kim, Kyung Sik Yi, Chi-Hoon Choi, Sang-Hoon Cha, Chul-Kee Park, Ja-Lok Ku

**Affiliations:** 1 Korean Cell Line Bank, Laboratory of Cell Biology, Cancer Research Institute, Seoul National University College of Medicine, Seoul, 03080 Republic of Korea; 2 Cancer Research Institute, Seoul National University, Seoul, 03080 Republic of Korea; 3 Ischemic/Hypoxic Disease Institute, Seoul National University College of Medicine, Seoul, 03080 Republic of Korea; 4 Department of Neurosurgery, Seoul National University Hospital and Seoul National University College of Medicine, Seoul, 03080 Republic of Korea; 5 Department of Radiology, Chungbuk National University Hospital and Chungbuk National University College of Medicine, Cheongju, Chung Buk 28644 Republic of Korea; 6 Chungbuk National University College of Medicine, Cheongju, Chung Buk 28644 Republic of Korea; 7 Department of Biomedical Sciences, Seoul National University College of Medicine, Seoul, 03080 Korea

**Keywords:** Genetic databases, CNS cancer

Correction to: *Scientific Data* 10.1038/s41597-023-02365-y, published online 12 July 2023

In this article, the grant number NRF2022R1A5A102641311 relating to National Research Foundation of Korea was omitted. The original article has been corrected.

