# Supplementary material for: Patient-derived glioblastoma cell lines with conserved genome profiles of the original tissue
Source: Sci Data. 2023 Jul 12;10:448. doi: 10.1038/s41597-023-02365-y (PMC10338444; doi:10.1038/s41597-023-02365-y)
Supplement: Supplementary file 1 — Supplementary Information [file 41597_2023_2365_MOESM1_ESM.docx]

**Supplementary Information**

**Patient-derived glioblastoma cell lines with conserved genome profiles of the original tissue**


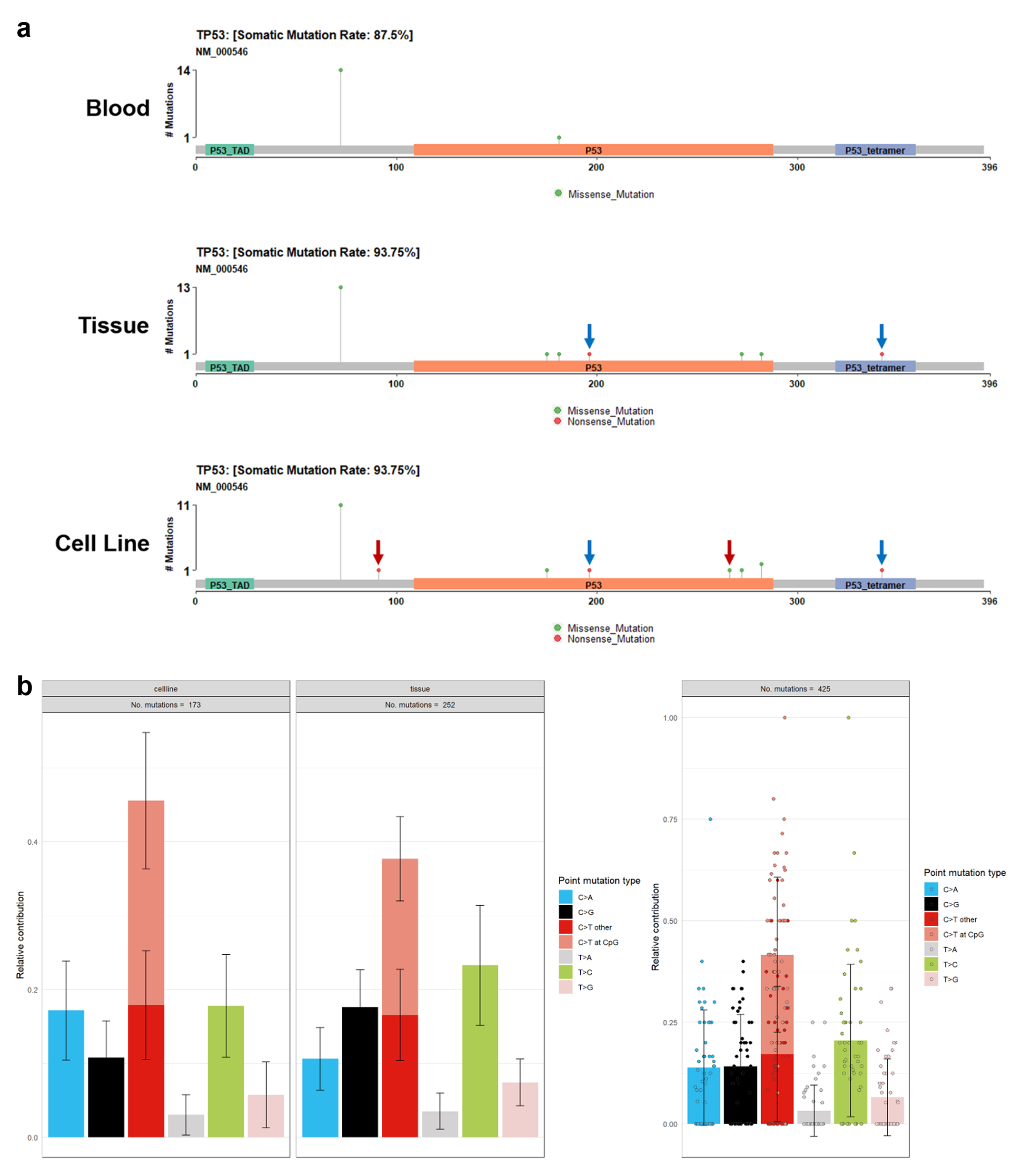


**Supplementary Fig 1a.** Lollipop plot of TP53 indicates that the nonsense mutation pointed by blue arrows exclusively presented at the tissue and cell line samples, which implied these are the potential driver mutations. The missense mutation and nonsense mutations highlighted by red arrows were harbored by cell lines only. **b.** Mutational concordance analysis shows that the relative contribution of point mutation type of the original tumor tissue was conserved in the cell lines.


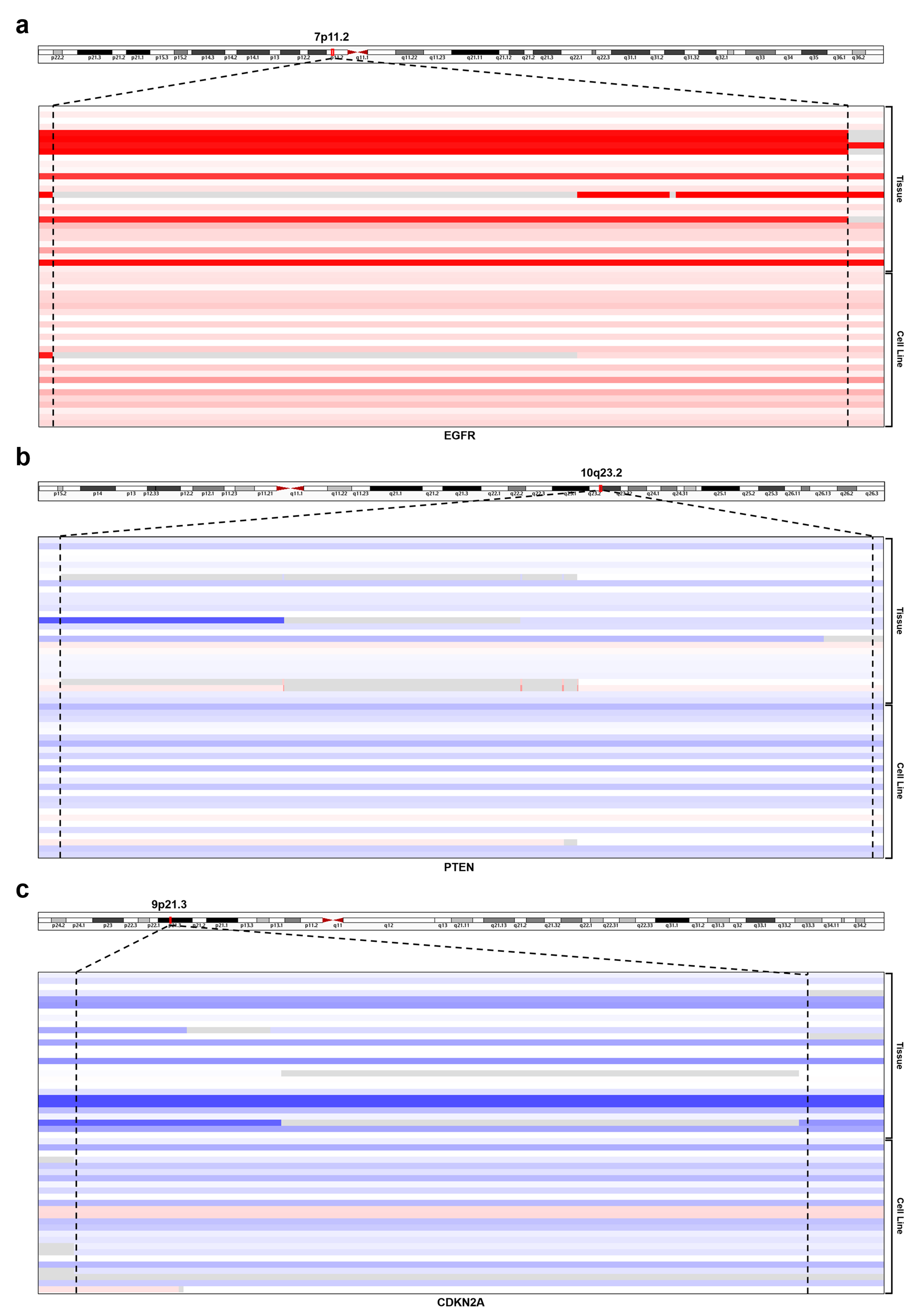


**Supplementary Fig 2a.** Frequently amplified region (7p11.2) in Glioblastoma harboring EGFR was inspected in our samples. Majority of samples was amplified in the region. **b.** Moderate loss at 10q23.2 region harboring PTEN and **c.** 9p21.3 region harboring CDKN2A was detected.
